# Cytotoxicity of Selenium Immunoconjugates against Triple Negative Breast Cancer Cells

**DOI:** 10.3390/ijms19113352

**Published:** 2018-10-26

**Authors:** Soni Khandelwal, Mallory Boylan, Julian E. Spallholz, Lauren Gollahon

**Affiliations:** 1 Department of Nutritional Sciences, Texas Tech University, Lubbock, TX 79409, USA; soni.khandelwal@ttu.edu (S.K.); mallory.boylan@ttu.edu (M.B.); Julian.spallholz@ttu.edu (J.E.S.); 2 Department of Biological Sciences, Texas Tech University, Lubbock, TX 79409, USA

**Keywords:** TNBC cells, HME50-5E cells, selenium, selenotrastuzumab, selenobevacizumab, sodium selenite, trastuzumab, bevacizumab, reactive oxygen species, targeted immunotherapies

## Abstract

Within the subtypes of breast cancer, those identified as triple negative for expression of estrogen receptor α (ESR1), progesterone receptor (PR) and human epidermal growth factor 2 (HER2), account for 10–20% of breast cancers, yet result in 30% of global breast cancer-associated deaths. Thus, it is critical to develop more targeted and efficacious therapies that also demonstrate less side effects. Selenium, an essential dietary supplement, is incorporated as selenocysteine (Sec) in vivo into human selenoproteins, some of which exist as anti-oxidant enzymes and are of importance to human health. Studies have also shown that selenium compounds hinder cancer cell growth and induce apoptosis in cancer cell culture models. The focus of this study was to investigate whether selenium-antibody conjugates could be effective against triple negative breast cancer cell lines using clinically relevant, antibody therapies targeted for high expressing breast cancers and whether selenium cytotoxicity was attenuated in normal breast epithelial cells. To that end, the humanized monoclonal IgG1 antibodies, Bevacizumab and Trastuzumab were conjugated with redox selenium to form Selenobevacizumab and Selenotrastuzumab and tested against the triple negative breast cancer (TNBC) cell lines MDA-MB-468 and MDA-MB-231 as well as a normal, immortalized, human mammary epithelial cell line, HME50-5E. VEGF and HER2 protein expression were assessed by Western. Although expression levels of HER2 were low or absent in all test cells, our results showed that Selenobevacizumab and Selenotrastuzumab produced superoxide (O_2•_−) anions in the presence of glutathione (GSH) and this was confirmed by a dihydroethidium (DHE) assay. Interestingly, superoxide was not elevated within HME50-5E cells assessed by DHE. The cytotoxicity of selenite and the selenium immunoconjugates towards triple negative cells compared to HME-50E cells was performed in a time and dose-dependent manner as measured by Trypan Blue exclusion, MTT assay and Annexin V assays. Selenobevacizumab and Selenotrastuzumab were shown to arrest the cancer cell growth but not the HME50-5E cells. These results suggest that selenium-induced toxicity may be effective in treating TNBC cells by exploiting different immunotherapeutic approaches potentially reducing the debilitating side effects associated with current TNBC anticancer drugs. Thus, clinically relevant, targeting antibody therapies may be repurposed for TNBC treatment by attachment of redox selenium.

## 1. Introduction

Breast cancer is the fifth leading cause of cancer-related deaths world-wide with over 600,000 mortalities reported annually [1]. Within the subtypes of breast cancer, one stands out as highly aggressive, coupled with a triple negative histotype for estrogen receptor α (ESR1), progesterone receptor (PR) and human epidermal growth factor 2 (HER2) [2]. These triple negative breast cancers (TNBC) represent between 10% to 20% of all breast cancer (BC) cases and are responsible for ~30% of the BC associated deaths (reviewed in Saraiva et al., 2017) [3]. Along with its aggressive phenotype and heterogeneous nature, the greatest obstacle for successfully treating this disease is overcoming the lack of therapeutic targets due to its negative profile for PR, ESR1 and HER2 expression [4]. Targeted therapy for breast cancer (as well as other cancers) is directed towards overly expressed cellular receptors such as HER2, and ESR1 [5]. Monoclonal antibodies with binding affinity for cancer cell receptors block growth signals (e.g., Erbitux^®^; Herceptin^®^), prevent angiogenesis (e.g., Avastin^®^), deliver radioactive isotopes (e.g., Zevalin^®^) and chemotherapeutic drugs to cancer cells (e.g., Kadcyla^®^). While over-expression of these receptors has been considered essential for their use as targeted therapies, there has been little research to evaluate their use in cell lines that do not overexpress the receptors. Besides surgery and radiotherapy, systemic chemotherapy is the only TNBC treatment option for these patients and patient response to therapy and subsequent prognosis is known to be poor [6]. Standard therapy includes cocktails consisting of taxanes, anthracyclines and cyclophosphamide [7]. While more than 70 antibody-drug conjugates (ADCs) may be in various stages of clinical trials, none of the presently approved ADCs are targeted therapies for TNBC, making the need to identify more specific drugs critical.

Classification of BC histotype is currently based primarily on expression levels of ESR1, PR and HER2 [8]. ESR1 is considered positive if at least 1% of sampled cells are immunoreactive [9]. However, the threshold for expression levels determined to be viable for clinical relevance for HER2 status has recently been updated. Expression of HER2 is considered to have a positive threshold if >10%, with the fluorescent in situ hybridization HER2/CEP17 ratio of ≥2 is considered positive, or HER2 copy number is >6 signals per cell [10]. Based on their research, Korkaya and Wicha (2013) stated that breast cancer stem cells may be regulated by HER2, even in breast cancers that do not show amplification of the HER2 gene [11]. Importantly, it is NOT a total absence of HER 2 expression that constitutes a negative finding, and low levels of HER2 are present within the tumor cells. Thus, with the right “Smart-Bomb”, even low-level expression of HER2 may have utility for targeting TNBC while mitigating debilitating side effects associated with current chemotherapies.

Another important aspect of breast cancer classification that informs treatment decisions is the potential for developing metastases [12]. As our technology for dissecting key features of cancers continues to improve, new indicators of disease progression and outcome are discovered. For example, the importance of Vascular Endothelial Growth Factor (VEGF) for promoting angiogenesis [13]. In the case of cancer progression, VEGF expression promotes tumor vascularization [14], with the potential end result of metastasis. While Bevacizumab is no longer approved for breast cancer treatment, it is still used in colon and rectal cancer therapies [15]. The rationale for ceasing use in breast cancer treatments was due to lack of data indicating significant increased survival rates, improved quality of life, with a reduction of significant side effects [16]. However, the VEGF that is targeted by Bevacizumab has been identified by Diane et al. (2018) as an actionable target in triple negative breast cancers [17].

Could selenium be a type of “Metabolic Smart-bomb”? Selenium is dietarily essential and is incorporated as selenocysteine (Sec) into 25 human selenoproteins [18,19]. Selenoproteins such as glutathione peroxidase 1 (GPx1) through GPx4 and Thyrodoxine reductase exist as anti-oxidant enzymes and are of great importance to human health [20]. Studies have long demonstrated that elevated levels of selenium compounds are toxic [21,22]. Other studies have demonstrated that some selenium compounds, i.e., selenite [23] specifically hinder cancer cell growth and induce apoptosis in cancer cell culture models [21,22]. These experimental outcomes have triggered an in-depth interest in the mechanism by which Se can negatively impact cancer progression. There are numerous animal and human selenium studies that have concentrated on the role that selenium and its compounds and proteins have in cancer prevention, while less attention has been given to the inherent therapeutic toxic effects of selenium that may serve to treat cancers in vivo [24].

**Figure 1:**
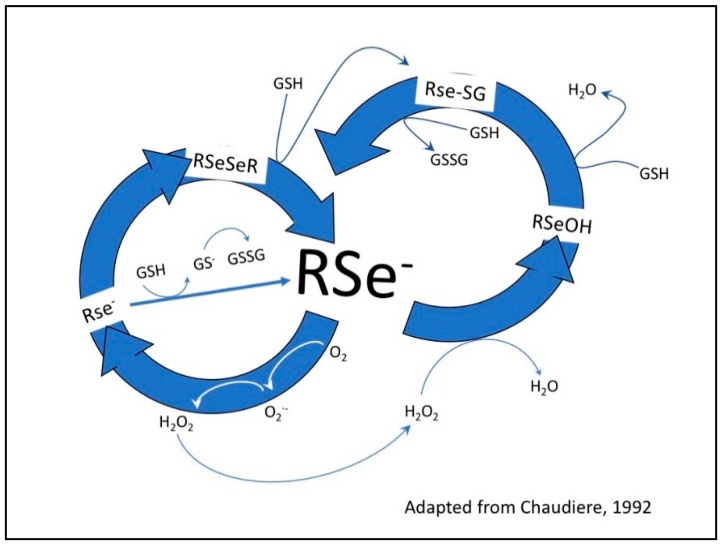
Redox cycling between selenium compounds and GSH. Glutathione (GSH) and other thiols reduce diselenides (RSeSeR) to selenenylsulfides (RSe-SG) and form the RSe^−^ a catalytic species. The one electron transfer from RSe^−^ to oxygen (O_2_) yields superoxide (O_2•_−) and a selenyl radical (RSe^−^). The superoxide is converted to hydrogen peroxide (H_2_O_2_) by superoxide dismutase. The hydrogen peroxide is reduced to water—this is the antioxidant property of glutathione peroxidase (GPx), forming a selenolate (RSeOH), which is associated with decay of the selenyl radical to diselenides. This happens at low concentrations of selenium. At high concentrations, RSe^−^ oxidizes GSH to oxidized glutathione (GSSG), producing superoxide, which in turn depletes the intracellular GSH concentration and the cell is subjected to oxidative stress, the basis of selenium toxicity.

Based on the ease with which selenium can be substituted for sulfur and the directed metabolic toxic effects of selenium, coupled with the high metabolism of cancer cells [25,26], we postulated that cancer cells would be much more sensitive to the effects of selenium at lower dosage levels than normal cells [23] (Figure 1). Furthermore, we hypothesized that clinically relevant antibodies could be utilized as vehicles to carry our redox selenium into the cancer cells and produce significantly more cell toxicity and death when compared to the normal cells. Finally, we were interested in observing whether selenium covalently conjugated to a receptor targeted antibody or ligand targeted antibody was more efficacious than native antibodies alone. To that end, we conjugated Trastuzumab (TZ) and Bevacizumab (BV) with the essential dietary nutrient selenium, in a redox active and toxic form using a selenium-modified Bolton-Hunter reagent. The therapeutic efficacy of repurposing clinically relevant off-target antibodies by selenium immunoconjugation in preclinical in vitro models of human TNBC is presented here.

## 2. Results

### 2.1. Analysis of Selenium in Control and Conjugated Antibodies

**Figure 2:**
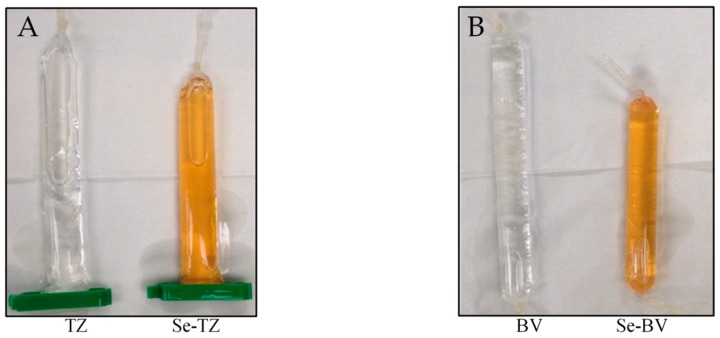
Dialyzed selenium-conjugated antibody products. After 72 h of conjugation time in pH 8.5 borate buffer after 72 h of dialysis against PBS pH 7.4, an orange color was observed for both selenium-antibody reactions. (**A**) Trastuzumab and Selenotrastuzumab. (**B**) Bevacizumab and Selenobevacizumab.

Redox selenium, as described in the Materials and Methods Section 4, was successfully conjugated to TZ and BV lysine residues using a modified Bolton-Hunter reagent. Conjugation resulted in an orange-red color in the final Se-antibody solution (Figure 2A,B).

The selenium concentrations as presented in Table 1 were computed to contain 32.12 µg Se/mg protein and 38.00 µg Se/mg protein respectively, for Se-TZ and Se-BV. The selenium concentrations of native TZ and BV were <0.007 µg Se/mg protein each. Table 1 shows the protein concentration of both native TZ or BV and Se-TZ or Se-BV following dialysis. The selenium concentration was determined by ICP-MS and the protein concentration was determined with a bicinchoninic acid (BCA) assay using bovine gamma globulin as a standard reference protein.

### 2.2. Homogenity and Stability of Selenium Immunoconjugates

**Figure 3:**
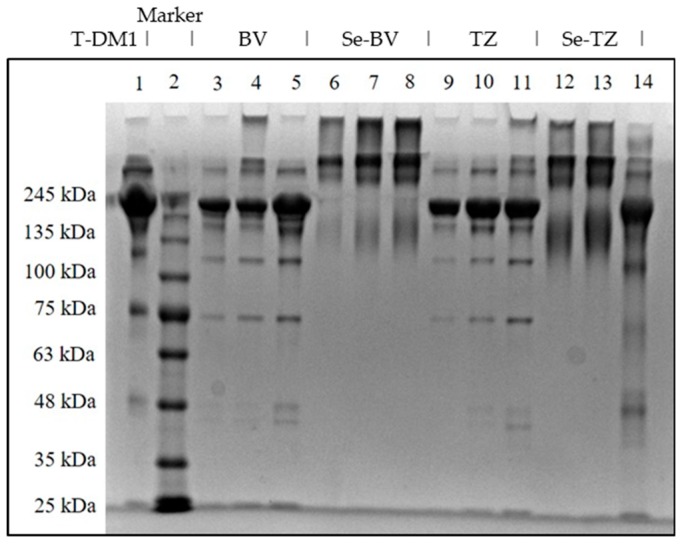
SDS-PAGE of Native and Se-conjugated mAbs under reducing conditions followed by Coomassie Blue R-250 staining. Lane 1: Kadcyla^®^ 20 µg; Lane 2: Marker; Lane 3: BV 5 µg; Lane 4: BV 10 µg; Lane 5: BV 20 µg; Lane 6: Se-BV 5 µg; Lane 7: Se-BV 10 µg; Lane 8: Se-BV 20 µg; Lane 9: TZ 5 µg; Lane 10: TZ 10 µg; Lane 11: TZ 20 µg, Lane 12: Se-TZ 10 µg; Lane 13: Se-TZ 20 µg; Lane 14: Gamma globulin 20 µg. (BV: Bevacizumab, Se-BV: Selenobevacizumab, TZ: Trastuzumab, Se-TZ: Selenotrastuzumab).

To assess the homogeneity of Se-TZ and Se-BV after conjugation with selenium; polyacrylamide gel electrophoresis (PAGE) of Se-labeled and native TZ or BV antibodies was performed (Figure 3). Both Se-immunoconjugates showed similarities in their migration patterns as did the native parent TZ and BV immunoglobulins. Separation of native TZ, native BV, Se-TZ and Se-BV were performed to evaluate the homogeneity of the heavy and light chains (Figure 3). High molecular weight bands above 245 kDa were observed in Se-TZ and Se-BV with Coomassie Blue staining, suggestive of new Se constructs. The electrophoretic patterns of Se-labeled and native TZ and BV antibodies were alike due to the loss of basic lysine residues from the selenium conjugation. T-DM1 (Kadcyla^®^), another mAb, was included as a control because of its similar backbone, consisting of TZ and linkers with three cytotoxic Emtansines [27]. T-DM1 is more electrophoretically similar to TZ than to Se-TZ.

**Figure 4:**
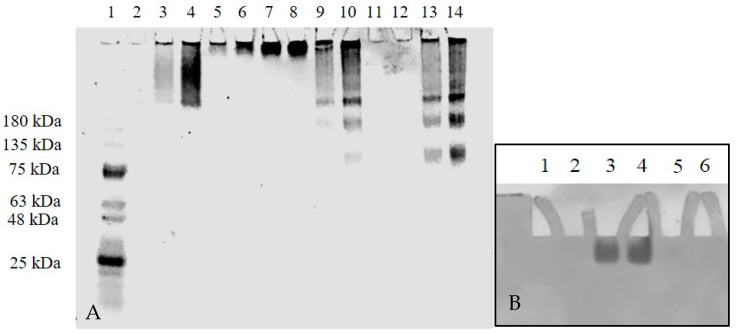
mAb migration on 4–20% Tris-Glycine PAGE gel under non-reducing conditions followed by Coomassie Blue R-250 stain. The image was taken using a Coomassie Blue filter. (**A**) Lane 1: molecular marker; Lane 2: purified human IgG 10 µg; Lane 3: bovine gamma globulin 5 µg; Lane 4: bovine gamma globulin 10 µg; Lane 5: T-DM1 5 µg; Lane 6: T-DM1 10 µg; Lane 7: BV 5 µg; Lane 8: BV 10 µg; Lane 9: Se-BV 5 µg; Lane 10: Se-BV 10 µg; Lane 11: TZ 5 µg; Lane 12: TZ 10 µg; Lane 13: Se-TZ 5 µg; Lane 14: Se-TZ 10 µg. (**B**) TZ migration on 4–20% Tris-Glycine PAGE gel under non-reducing conditions with electrophoretic poles reversed followed by Coomassie Blue R-250 stain. Lane 2: Ladder; Lane 3: TZ 5 µg; Lane 4: TZ 10 µg. (BV: Bevacizumab, Se-BV: Selenobevacizumab, TZ: Trastuzumab, Se-TZ: Selenotrastuzumab).

To assess other electrophoretic differences of the antibodies, at least two different concentrations In a second gel, two concentrations of TZ or BV were run on a 4–20% Tris-Glycine polyacrylamide gel electrophoresis (PAGE) under non-reducing conditions (Figure 4A). A trailing band was seen for Se-TZ and Se-BV similar to the trailing band of bovine gamma globulin. The results of Se-TZ and Se-BV show that the banding pattern was independent of the sample volume loaded across all the different samples. When no migration was seen for TZ in lanes 11 and 12 (Figure 4A), two concentrations of TZ and a protein ladder were loaded onto a 4–20% Tris-Glycine Gradient PAGE and run under non-reducing conditions with the electrophoretic migration pole charges reversed. TZ protein expression was then observed (Figure 4B).

### 2.3. Detection of Superoxide In Vitro and In Situ

**Figure 5:**
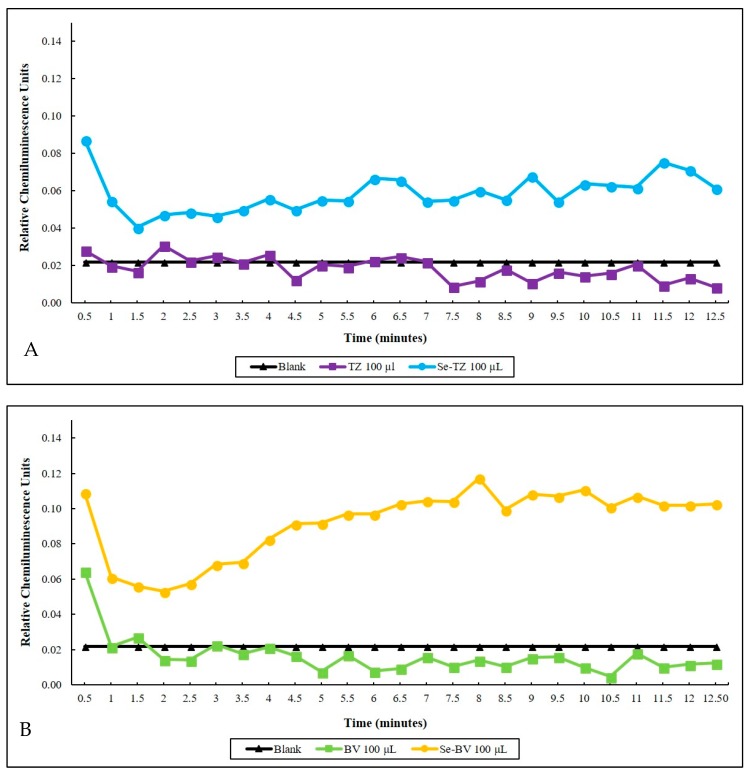
Time-dependent superoxide generation as measured by lucigenin chemiluminescence (CL). (**A**) Blank 100 µL Trastuzumab and 100 µL of Selenotrastuzumab = 8.8 µg of Se were compared. Proteins alone were further suppressive of the background (Blank CL) due to the slow auto-oxidation of GSH. (**B**) Blank 100 µL Bevacizumab and 100 µL Selenobevacizumab = 8.1 µg of Se. Proteins and BV alone were further suppressive of the background (Blank CL) due to the slow auto-oxidation of GSH.

Selenite, the selenium immunoconjugates and native mAbs were analyzed by a Lucigenin superoxide chemiluminescence (CL) assay. The CL detection cocktail consisted of 500 µL of 1 mg/mL GSH and 20 µg/mL of Lucigenin in a PBS buffer, pH 7.4. with selenite generating superoxide in a concentration-dependent manner. Over a 12.5 min CL counting period, the superoxide generated by the two selenium immunoconjugates in the presence of glutathione (GSH) shown in real-time was seen to be higher than native TZ (Figure 5A) or BV (Figure 5B) at the same GSH concentration. Native proteins tend to quench the small amount of CL from the superoxide generated from the auto-oxidation of GSH in control assays. The Se-TZ and Se-BV accelerated the oxidation of GSH, generating greater amounts of superoxide and CL, accounting for the immunoconjugates’ toxicity.

**Figure 6:**
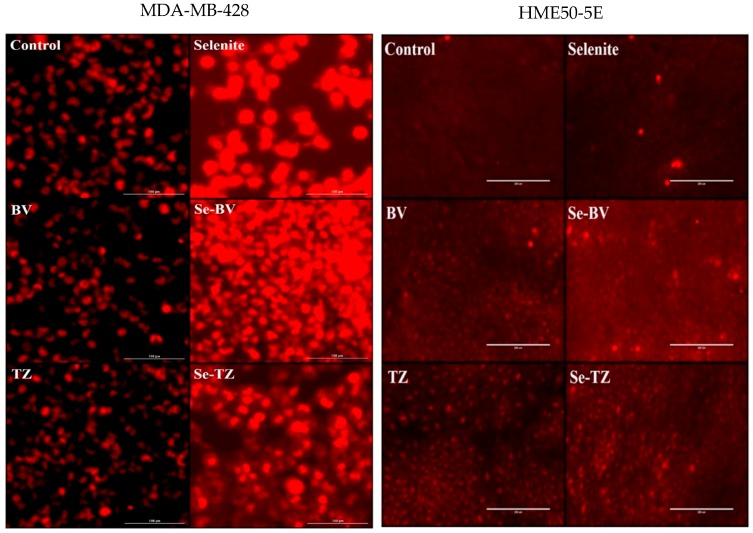
Photomicrographs of intracellular superoxide generation by DHE red florescence from MDA-MB-468 Cells (Left Panels) and HME50-5E cells (Right Panels). Results were photographed after 30 min of treatment with Control, selenite (10 µg Se), BV (153.7 µg protein), Se-BV (10 µg Se and 153.7 µg protein), TZ (131.1 µg protein), and Se-TZ (10 µg Se and 131.1 µg protein).

Additionally, generation of superoxide (O_2•_−) anions by selenite and the selenium immunoconjugates in situ was analyzed by DHE (Figure 6). Results showed the much higher intracellular production of superoxide (O_2•_−) anions in MDA-MB-468 cells by Se-TZ, Se-BV and selenite over control and native TZ or BV-treated cells (Figure 6). The CL and DHE results collectively suggest the ability of Se-TZ or Se-BV to generate greater amounts of intracellular superoxide presumably from the oxidation of glutathione and other thiols within the TNBC cells. However, intensity of the DHE signal indicated superoxide generation was significantly less in HME50-5E cells (Figure 6) treated with Se-TZ or Se-BV.

### 2.4. Evaluating the Cytotoxicity of the Se-Immunoconjugates

**Figure 7:**
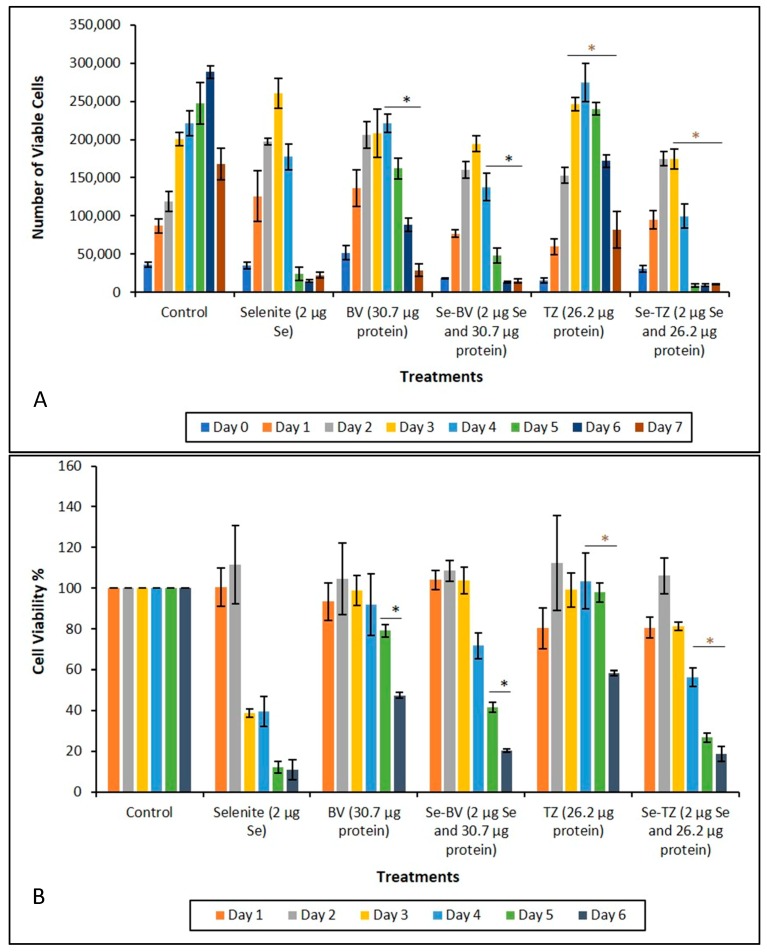
Analysis of cytotoxicity as a function of percent cell viability during treatment. (**A**) Cytotoxic effects of control, selenite, BV, Se-BV, TZ and Se-TZ Treatments against MDA-MB-468 cells. Forty-thousand cells were seeded in 48-well-plates and treated (Day 0 of treatment). Viable cells were counted for 7 days and analyzed by Trypan Blue exclusion. The data is expressed as Means ± SE (*n* = 3). Statistical treatments were compared using two sample *t*-tests. Treatments were considered statistically significant if *p* ≤ 0.05 and indicated by * (brown color). (**B**) Growth inhibition of control, selenite, BV, Se-BV, TZ, or Se-TZ treated MDA-MB-468 cells as determined by MTT assay over 6 days. Forty-thousand cells were seeded in 48-well-plates and treated (Day 0 of treatment). The data is expressed as the Means ± SE (*n* = 3). Treatments were compared using two sample *t*-tests. Treatments were considered statistically significant if *p* ≤ 0.05 (represented by * (black color)). Asterisks indicate significant differences between TZ and Se-TZ (**A**) and BV and Se-BV (**B**).

Incubation of both TNBC cell lines MDA-MB-468 and MDA-MB-231 (data similar to MDA-MB-468, not presented for simplification) with Se-TZ or Se-BV for a treatment period of 0–7 days, resulted in significant decreases in viable cell numbers for both immunoconjugates over their respective controls, TZ and BV treatments. Results are shown in Figure 7A and were determined using Trypan Blue exclusion in a Beckman Vi-Cell Particle Characterization Unit. Since Trypan Blue exclusion is based on cell membrane integrity, the results suggest that the effect of the Se-immunoconjugates on the TNBC cells perturbs membrane integrity.

The MTT Formazan assay for the MDA-MB-468 cells demonstrated that the Se-Immunoconjugates were cytotoxic over their respective native mAbs, TZ and BV, in a time-dependent manner. The results (Figure 7B) indicate the effects of Se-immunoconjugates on MDA-MB-468 cells are due to a loss of membrane integrity. ANOVA results for these experiments are shown in Table 2, Table 3, Table 4 and Table 5. Level of significance was determined at *p* ≤ 0.05 and is highlighted in yellow.

**Figure A1:**
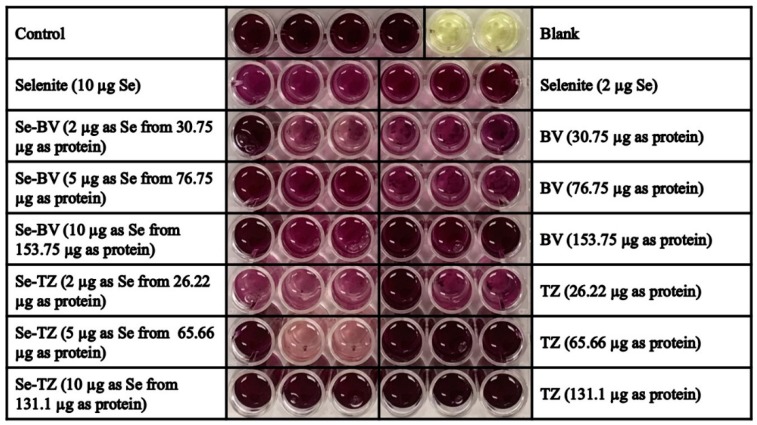
HME50-5E MTT Plate Photo on Day 3 Post Treatment showing the insoluble formazan salt.

**Figure A2:**
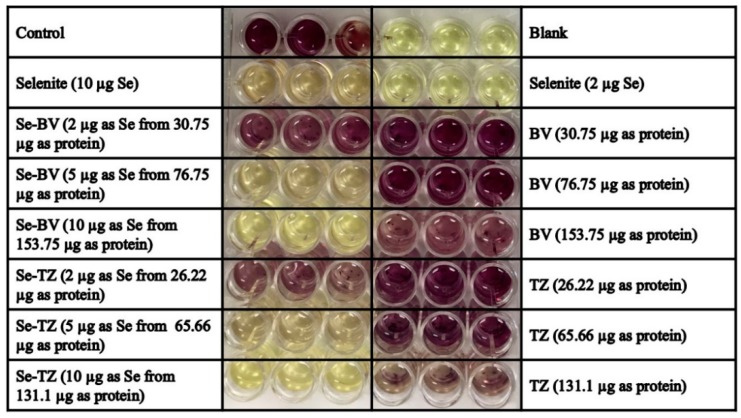
HME50-5E MTT Plate Photo on Day 4 Post Treatment showing the insoluble formazan salt.

**Figure A3:**
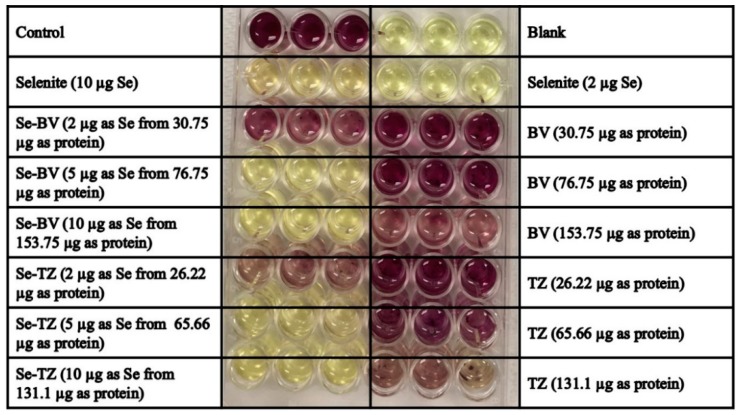
HME50-5E MTT Plate Photo on Day 5 Post Treatment showing the insoluble formazan salt.

**Figure A4:**
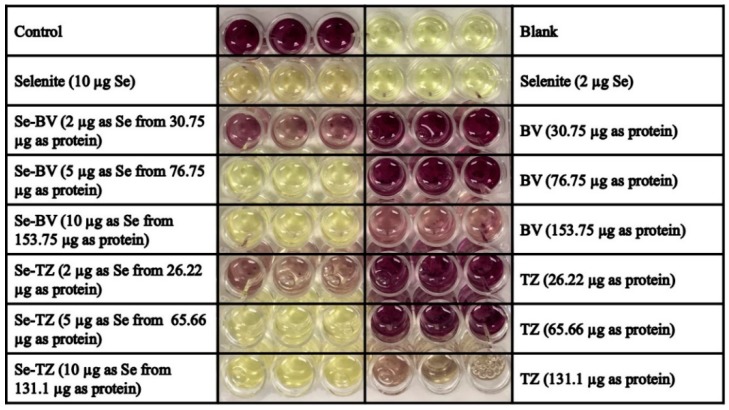
HME50-5E MTT Plate Photo on Day 6 Post Treatment showing the insoluble formazan salt.

Interesting and of significance were the observations that the HME50-5E cells adhered so strongly to the Corning well-plates that collection numbers obtained with either Trypsin/EDTA or Accutase^®^ were not reflective of actual cell numbers because many cells that remained, adhered to the plate. When the cells were finally removed, effects due to treatment or harvesting conditions could not be differentiated. However, MTT assay analysis performed on the HME50-5E cells determined that they were highly metabolically active following treatments with Se-TZ and Se-BV. The MTT assay results indicated that not only were the HME50-5E cells treated with Se-immunoconjugates metabolically functional, but also that the Formazan readings were greater than the upper range for instrumental resolution at 570 and 690 nm (Figure A1, Figure A2, Figure A3 and Figure A4) in stark contrast to the TNBC results.

### 2.5. Morphological Changes in Triple Negative Breast Cancer (TNBC) Cells and HME50-5E Cells after Se-Trastuzumab (TZ) or Se-Bevacizumab (BV) Treatment

**Figure 8:**
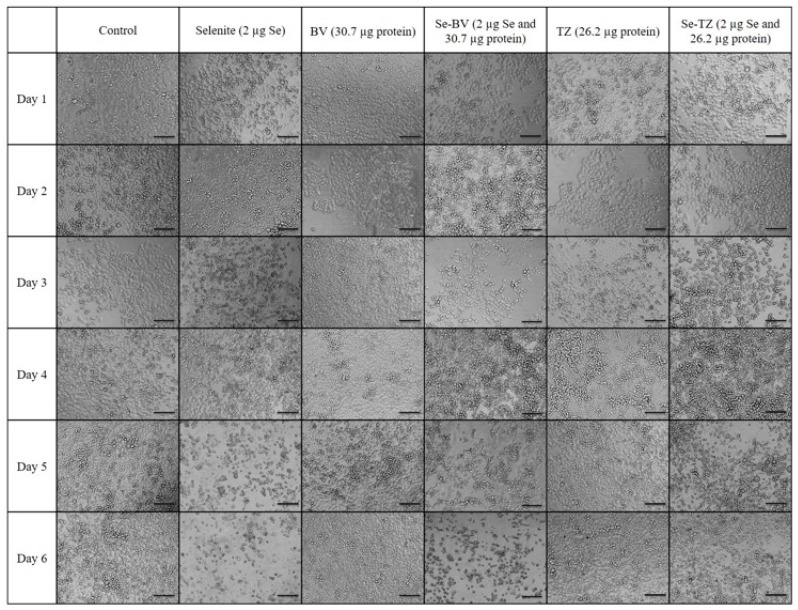
Photomicrographs of the morphological changes observed in control, Selenite, Bevacizumab (BV), Selenobevacizumab (Se-BV), Trastuzumab (TZ) and Selenotrastuzumab-Treated MDA-MB-468 Cells. Treatment of the MDA-MB-468 Cells with Selenite, Se-BV and Se-TZ revealed morphological changes indicative of membrane disruption and decreased cell viability in comparison to the native antibodies BV and TZ. Representative fields of view of MDA-MB-468 cells. Cells were photographed under phase contrast conditions at 20× magnification. Scale bar = 100 µm.

**Figure 9:**
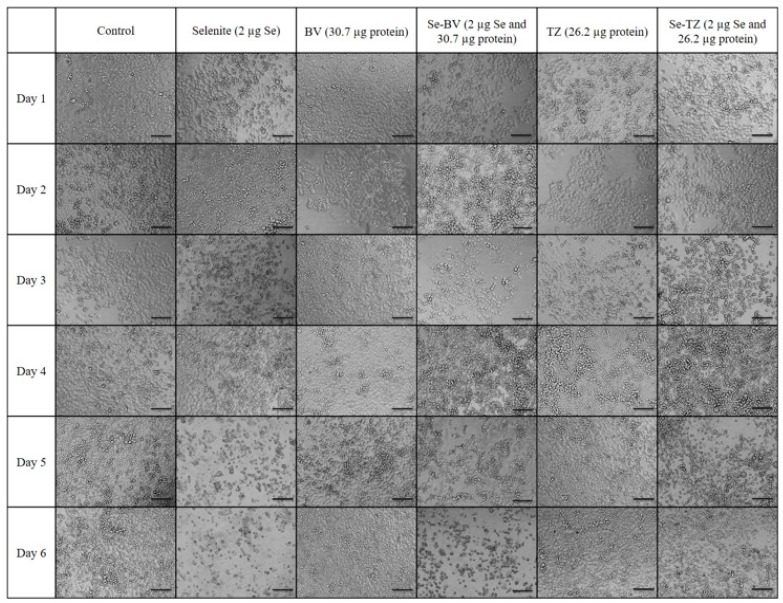
Photomicrographs of the morphological changes observed in control, Selenite, Bevacizumab (BV), Selenobevacizumab (Se-BV), Trastuzumab (TZ) and Selenotrastuzumab-treated HME 50-5E Cells. Treatment of HME 50-5E cells with Selenite, Se-BV and Se-TZ did not induce severe morphological cell changes in comparison to those seen in the selenium-treated MDA-MB-468 cells. Representative fields of view of HME50-5E cells. Cells were photographed under phase contrast conditions at 20× magnification. Scale bar = 100 µm.

While informative, the MTT and Trypan Blue assays do not distinguish between the reduction in cell number nor a decline in metabolic function, respectively. Thus, the MDA-MB-468 cells were visually assessed and morphological effects were documented with light microscopy after incubation with Se-TZ or Se-BV. To visualize the cells, morphological effects to the varied treatments, TNBC cells and HME50-5E cells were treated with equal amounts of selenium as selenite, Se-TZ or Se-BV. Images were generated with an Olympus IX71 inverted microscope at 20× magnification and representative photographs are shown for MDA-MB-468 (Figure 8) and HME 50-5E treated cells (Figure 9). The MDA-MB-468 cells and HME50-5E cells are both adherent epithelial cells. With adherent cells, detachment from the substratum as well as morphological change is indicative of apoptosis [28]. Selenite, Se-TZ or Se-BV-treated MDA-MB-468 cells all show gross cell morphological change, irregular or rounder cells, apparent growth inhibition with cell swelling or shrinking. It is likely that cell growth is inhibited by the redox selenium and cells detach from the culture substratum. Additionally, the cell morphology in the MDA-MB-468 cells changes from grape-like cell clusters [29] to the extensive cytoplasmic vacuolization that becomes even more dramatic over time. Similar morphological changes in cancer cells treated with other redox active Se compounds have been previously reported [21]. The HME50-5E cells visually appear mildly stressed. However, confluency and cell morphology were largely maintained in HME50-5E cells. Furthermore, the onset of these morphological changes was time-dependent. Obvious morphology changes were not observed until Day 4, whereas some changes were evident in the TNBC cells after 24 h. Moreover, the HME50-5E cells appeared resistant to sodium selenite treatment throughout this experiment. These findings suggest selenium is much less cytotoxic to these more normal cells. Alternatively, the addition of selenium to the antibody sensitizes the cancer cells to treatment. From an in vivo therapeutic point of view, these observations suggest that Se-TZ or Se-BV may be more effective in suppressing tumor growth than their native analogs.

### 2.6. Selenium Treatment Induced Apoptosis

**Figure 10:**
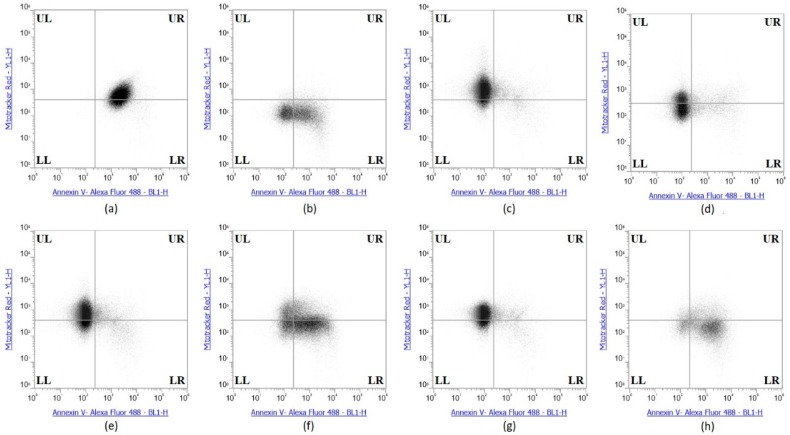
Se-Immunoconjugates induced greater apoptosis than native mAbs in MDA-MB-468 Cells. (**a**) Sutent (apoptosis control), (**b**) 0.1% Triton X-100 (necrosis control), (**c**) Control untreated (**d**) Selenite, (**e**) Bevacizumab, (**f**) Selenobevacizumab, (**g**) Trastuzumab, (**h**) Selenotrastuzumab Treatments. MDA-MB-468 cells were stained with Annexin V/MitoTracker Red and subjected to flow cytometric analysis. The four quadrants represent—living cells (Upper Left; UL: Annexin V, MitoTracker Red), early apoptotic (Upper Right; UR: Annexin V, MitoTracker Red), late apoptotic (Lower Right; LR: Annexin V, MitoTracker Red) or necrotic (Lower Left; LL: Annexin V, MitoTracker Red) stages.

The collective experimental results show that Se-immunoconjugates, Se-BV and Se-TZ are very likely generating intracellular O_2•_− from GSH and other thiol oxidation with treated cells losing their membrane and metabolic integrity. There is reduction in mitochondrial activity and finally detachment from the substratum; all events cumulatively inhibit MDA-MB-468 cell proliferation initiated by superoxide generation-induced apoptosis. In contrast, the cytotoxic effects of selenium are, by these measures, significantly less on the HME50-5E cells. To confirm the mechanism of cell death, MDA-MB-468 cells were stained with Annexin V and Mitotracker Red and analyzed by flow cytometric analysis (Figure 10). Positive controls included Sutent for apoptosis and Triton X-100 for necrosis. The flow cytometry data showed that the selenite treatments predominantly resulted in cell necrosis and the Se-TZ and Se-BV-induced cytotoxicity was primarily due to apoptosis in the TNBC cells. Native antibodies, TZ or BV were not observed to induce either necrotic or apoptotic effects.

**Figure 11:**
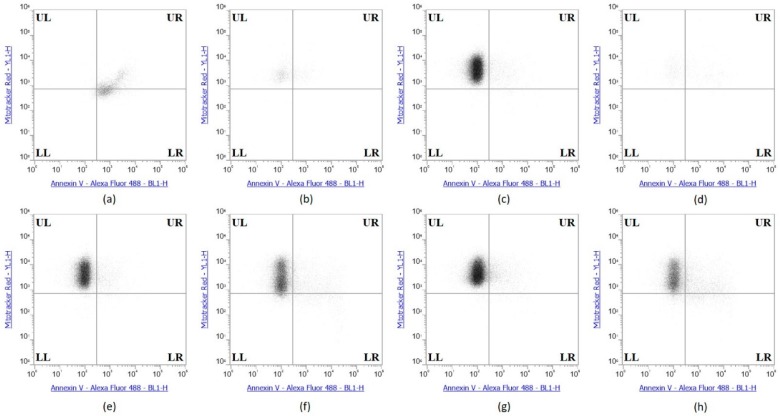
Se-Immunoconjugates induced no apoptosis in HME50-5E Cells. (**a**) Sutent (apoptosis control), (**b**) 0.1% Triton X-100 (necrosis) control), (**c**) Control untreated, (**d**) Selenite, (**e**) Bevacizumab, (**f**) Selenobevacizumab, (**g**) Trastuzumab, (**h**) Selenotrastuzumab treatments in HME50-5E cells. HME50-5E cells were stained with Annexin V/PI Mitotracker Red and subjected to flow cytometric analysis. The four quadrants represent—living cells (Upper Left; UL: Annexin V, Mitotracker Red), early apoptotic (Upper Right; UR: Annexin V, Mitotracker Red), late apoptotic (Lower Right; LR: Annexin V, Mitotracker Red) or necrotic (Lower Left; LL: Annexin V, Mitotracker Red) stages.

**Figure 12:**
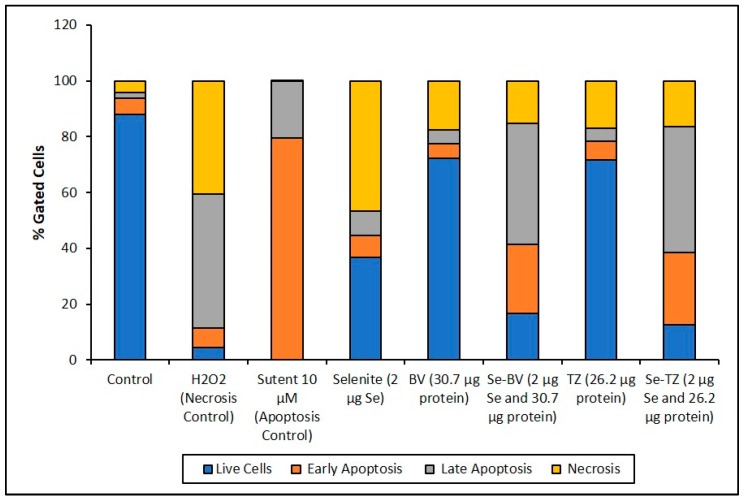
Cell representation (%) within the four quadrants for MDA-MB-468 cell treatments. Percent distribution of MDA-MB-468 apoptotic cells after treatment with H_2_O_2_, Sutent, Selenite as Se, Bevacizumab (BV), Selenobevacizumab (Se-BV), Trastuzumab (TZ), or Selenotrastuzumab (Se-TZ). Data is expressed as the Mean (*n* = 3).

**Figure 13:**
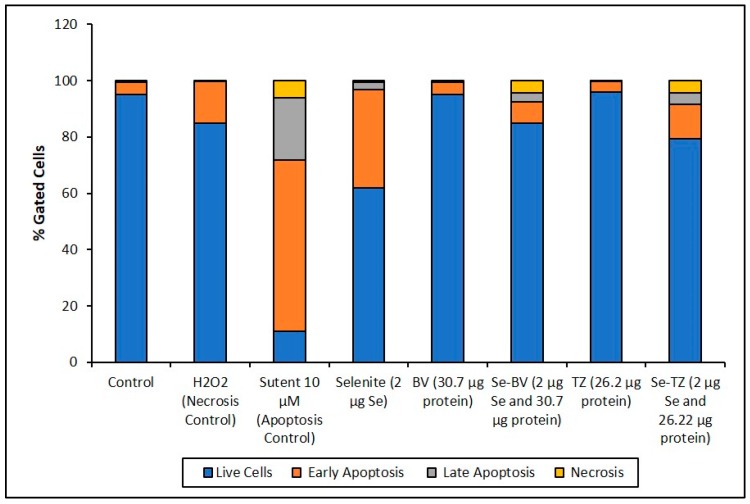
Cell representation (%) within the four quadrants for HME50-5E cell treatment. Percent distribution of HME50-5E apoptotic cells after treatment with H_2_O_2_, Sutent, Selenite as Se, Bevacizumab (BV), Selenobevacizumab (Se-BV), Trastuzumab (TZ) or Selenotrastuzumab (Se-TZ). Data is expressed as Mean (*n* = 3).

The Annexin V and MitoTracker Red Flow cytometry data for the TNBC cells (Figure 10) of just 2 µg Se as Se-TZ or Se-BV treatments showed a substantial proportion of the MDA-MB-468 cells transitioning from early to late apoptosis. The morphological phenotypes (Figure 8) of these apoptotic cells strongly correlate with the flow cytometry data. In contrast to the MDA-MB-468 cells, significant percentages of the HME50-5E cells were found in the upper left (UL) quadrant (Figure 11), corresponding to fully viable live cells. The total percentages of cells for various designated stages determined by flow cytometry are shown in Figure 12 and Figure 13.

To better appreciate the degree of apoptosis or necrosis in the TNBC cells versus the normal cells, the results are illustrated as stacked columns (Figure 12 and Figure 13). With this approach, it is much easier to observe the striking differences between Se-TZ or Se-BV-induced apoptosis laterally (in comparison to the native mAb treatment with TZ or BV) in the TNBC (Figure 12). Additionally, longitudinal differences between cytotoxicity and apoptosis-induced pathways are observed between the TNBC cells (Figure 12) and the HME50-5E (Figure 13) for Se-TZ and Se-BV treatments.

### 2.7. Human Epidermal Growth Factor 2 (HER2) and Vascular Endothelial Growth Factor (VEGF) Protein Expression

**Figure 14:**
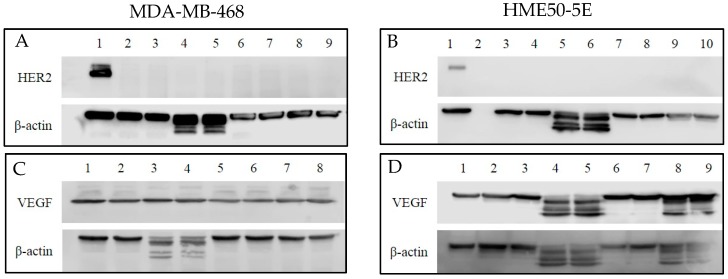
Western blot analysis of the expression level of human epidermal growth factor 2 (HER2) and vascular endothelial growth factor (VEGF) in MDA-MB-468 and HME50-5E cells treated with Selenite, Trastuzumab (TZ) and Selenotrastuzumab (Se-TZ). Total cell lysates were subjected to SDS-PAGE followed by Western blotting. Membranes were probed with the anti-HER2, anti-VEGF, or anti β-actin antibodies followed by peroxidase conjugated rabbit anti-mouse antibodies and visualization was performed by the enhanced chemiluminescence (ECL) detection system. (**A**) HER2 and MDA-MD-468 cells. Lane 1: BT474 as HER2 positive loading control; Lanes 2 and 3: Control; Lanes 4 and 5: Selenite (2 µg as Se) treatments; Lanes 6 and 7: TZ (26.22 µg as protein) treatments; Lanes 8 and 9: Se-TZ (2 µg as Se from 26.22 µg as protein) treatments. (**B**) HER2 and HME50-5E. Lane 1: BT474 as HER2 positive loading control; Lane 2: Molecular weight markers; Lanes 3 and 4: Control; Lanes 5 and 6: Selenite (2 µg as Se) treatments; Lanes 7 and 8: TZ (26.22 µg as protein) treatment; Lanes 9 and 10: Se-TZ (2 µg as Se from 26.22 µg as protein) treatments. (**C**) VEGF and MDA-MB-468 cells. Lanes 1 and 2: Control; Lanes 3 and 4: Selenite (2 µg Se) treatments; Lanes 5 and 6: BV (30.7 µg protein) treatment; Lanes 7 and 8: Se-BV (2 µg Se from 30.7 µg protein) treatments. (**D**) VEGF and HME50-5E cells. Lane 1: MDA-MB-468 as VEGF positive loading control; Lanes 2 and 3: Control; Lanes 4 and 5: Selenite (2 µg Se) treatments; Lanes 6 and 7: BV (30.7 µg protein) treatment; Lanes 8 and 9: Se-BV (2 µg Se from 30.7 µg protein) treatments.

Since the primary targets for TZ and BV are HER2 and VEGF, respectively, it was important to establish baseline levels of protein expression (Figure 14A–D) in order to better understand the effects of selenium conjugation to these mAbs on the cells tested. To that end, Western blotting was used to detect their baseline protein expression levels in MDA-MB-468 and HME50-5E cells. BT-474 cell lysate was used as a positive loading control for immunoblotting and HER2 expression was detected at ~185 kDa. Following experimental treatments of cells, expression of HER2 in MDA-MB-468 (Figure 14A) and HME50-5E (Figure 14B) cells was not detected.

Baseline levels of VEGF remained consistent throughout treatment conditions for MDA-MB-468. VEGF was detected at ~23 kDa (Figure 14C). Cleaved VEGF expression was observed in HME50-5E cells following selenite treatment and Se-immunoconjugate treatment (Figure 14D). One possibility for cleaved VEGF expression in selenite treated cells is its non-specific, untargeted toxicity. An explanation for this observation is currently being explored.

β-actin was used as a loading control. Expression of β-actin was detected at ~42 kDa and cleaved β-actin expression was detected only after selenite treatment in both the cell lines (Figure 14A–D).

## 3. Discussion

The development and application of therapeutic monoclonal antibodies to treat cancers has become clinically more relevant as noted by the current extensive clinical use of Trastuzumab (Herceptin^®^), Bevacizumab (Avastin^®^), and Cetuximab (Erbitux^®^). Treatment of different cancers with these monoclonal antibodies individually require a “target” or protein receptor that is unique to the cancer or is overly-expressed by the cancer cell in proportion to normal cells. There are presently ~30 US Food and Drug Administration (FDA) approved monoclonal antibodies for the treatment of various medical conditions to include cancers. There are 70 or more monoclonal antibodies now in various stages of experimental or clinical development.

With the present cohort of clinical cancer antibodies in use, one criterion for FDA approval is the extension of the life span of cancer patients. Life extension with monoclonal antibody treatment averages a few months for many patients with their primary cancer relapsing due to the development of monoclonal antibody resistance, i.e., failure of mAbs to control cancer growth. Resistance to monoclonal antibody therapy is not uncommon within an 18 to 24-month period after diagnosis and the commencement of initial treatment. To overcome the resistance to primary treatment, pharmaceutical companies have begun to add a chemotherapeutic drug to their “naked” monoclonal antibody. The “naked” monoclonal antibody conjugated with a therapeutic drug is often referred to as an antibody drug conjugate, or ADC. ADC applications are expanding to help counter primary antibody treatment relapse. The best example of this therapeutic approach to relapse is with the conversion of Trastuzumab (Herceptin^®^) to ado-Ematansine (Kadcyla^®^) also known as T-DM1. T-DM1 carries several copies of covalent Ematansine, a chemotherapeutic drug that interferes with the endocellular cytoskeletal formation prior to cell division. The T-DM1 originating from Trastuzumab retains its monoclonal targeting ability for the HER2 receptor. T-DM1 is the first of its kind of ADCs to be approved by the FDA for clinical use against HER2 positive, Trastuzumab-resistant breast cancers.

Triple Negative Breast Cancer (TNBC) occurs in 10–20% of all breast cancers globally and is devoid of any presently known “targets” or receptors against which small drugs or monoclonal antibodies may be developed. Chemotherapy with taxanes, anthracyclines and cyclophosphamides are the only course of TNBC treatment, and the prognosis and the outcomes of patient treatments is poor.

The rationale for investigating the potential of selenium conjugation to form a “Metabolic Smart-Bomb” is based on the well-established redox chemistry of selenium by Seko et al. [30] for selenite and later by Chaudiere et al., [31] for reduced diselenides.

Our laboratory has been working on conjugation methods to improve the cytotoxicity of “naked” monoclonal antibodies with redox selenium forming ADCs with a toxic redox specie of selenium. This was first accomplished with Trastuzumab (Herceptin^®^) and was in vitro shown to be more efficacious in stopping the growth of a Trastuzumab resistant cancer cell line, JIMT-1 than either Trastuzmab or TDM-1 [32]. Here, we approached the possibility that selenium ADC’s of Trastuzumab (Herceptin^®^) and/or Bevacizumab (Avastin^®^) might be effective in stopping in vitro cell growth of two TNBC cell lines MDA-MB-468 and MDA-MB-231. The response of these two TNBC cell lines with the two native monoclonal antibodies and their selenium ADCs was compared to an immortalized, normal epithelial cell line, HME50-5E.

In the present study, an organic selenocyanate was attached to TZ and BV, using a selenium modified Bolton-Hunter reagent, forming redox active Se-immunoconjugates. The concentration of selenium in these newly developed immunoconjugates was confirmed by ICP-MS. Analysis of in vitro superoxide reactions using lucigenin-chemiluminescence confirmed the generation of superoxide by the oxidation of GSH by selenite as previously shown by Seko et al., [30] and more importantly to Se-TZ and Se-BV but not in the CL cocktail with TZ or BV alone. Fluorescence microscopy confirmed the intracellular generation of superoxide in situ by these Se-immunoconjugates in the cancer cells. Similar results have been reported from prior studies comparing the cytotoxicity of selenite, selenate, selenocystine and selenomethionine towards the human mammary tumor cell line HTB123/DU4475 in vitro [33].

Inhibition of cell survival was examined by Trypan Blue exclusion starting immediately after treatment until Day 7 post treatment. The experimental treatments show that selenite, Se-TZ or Se-BV treated cells exhibit a general loss of viable cells over time as measured against control cells. To better understand the mechanism of cell death demonstrated by the Trypan Blue exclusion results, an alternative cytotoxic assessment assay of cell viability, i.e., the MTT assay (3-[4,5-dimethylthioazol-2-yl]-2-5-diphenyltetrazolium bromide) was performed, where the tetrazolium salt ring was cleaved by viable cells forming the insoluble Formazan. This method would also help address the question of cell viability in the cells when treated with Se-TZ or Se-BV. Our results showed that incubation of MDA-MB-468 cells with Se-TZ or Se-BV resulted in significant reduced proliferation by the MTT assay. These experimental results also appear to be confirmed by the optical assessment of altered cell morphology and detachment from substratum as photographed and shown in Figure 7 and Figure 8. This is indicative of apoptosis in the case of adherent cells [28]. Previous studies have established that selenium induces apoptosis by the intrinsic (mitochondrial-mediated) pathway [34,35,36,37]. Furthermore, apoptosis programmed cell death is associated with morphological, biochemical and molecular changes occurring in the cell [38]. From the results of Trypan Blue and MTT assays, we knew that the Se-immunoconjugates had an effect on cell membranes and mitochondrial activity.

A loss of mitochondrial membrane potential (ΔψM) indicates the loss of cell viability as the electrical potential reflects the pumping of protons across the inner mitochondrial membrane during the processes of electron transport and oxidative phosphorylation that drives the conversion of ADP to ATP [39]. Therefore, MDA-MB-468 and HME50-5E cells were stained with MitoTracker Red CMXRos (instead of Propidium Iodide), which stains mitochondria in live cells and its accumulation is dependent upon the cell’s ΔψM. In this study, the ΔψM was measured by flow cytometry and the results demonstrated that a dose of 2 µg Se as Se-TZ or Se-BV caused a decrease in the ΔψM potential and increased the percent apoptotic MDA-MB-468 cells (Figure 10 and Figure 12). In contrast, there was no change in the ΔψM (UL quadrant) of control, Se-TZ or Se-BV-treated HME50-5E cells (Figure 11 and Figure 13), indicating viable cell populations. Hence, HME50-5E cells do not undergo apoptosis following Se-TZ or Se-BV treatment. These observations again suggest the probable role of selenium-induced oxidative stress/glutathione triggered apoptosis associated with both increased generation of superoxide and a decrease in the level of the mitochondrial membrane potential. Studies have reported that modulation of mitochondrial functions to regulate apoptosis is one of the most affected pathways of Se compounds in cancer therapy [40].

The results from Western blot analysis show cleaved β-actin bands for selenite treatment in MDA-MB-468 cells and HME50-5E cells. These may have some biological and therapeutic implications like cell-cell interactions, cell migration (metastasis) and proliferation [41]. This interesting observation requires additional investigation and was outside the scope of this current project. Furthermore, the safety and efficacy of sodium selenite has been evaluated in a Phase I clinical trial. The results from this trial show that no major toxicity was reported when 10.2 mg/M^2^ of sodium selenite was administered IV to cancer patients [42].

Due to its apoptotic activity, Sutent (Pfizer) was used as a positive control for the induction of apoptosis in TNBC [43] and HME50-5E cells. Interestingly, Sutent demonstrated even greater potency in killing the normal cells. To our knowledge, this is the first report of any toxicity of Sutent on normal mammary epithelial cells.

**Figure 15:**
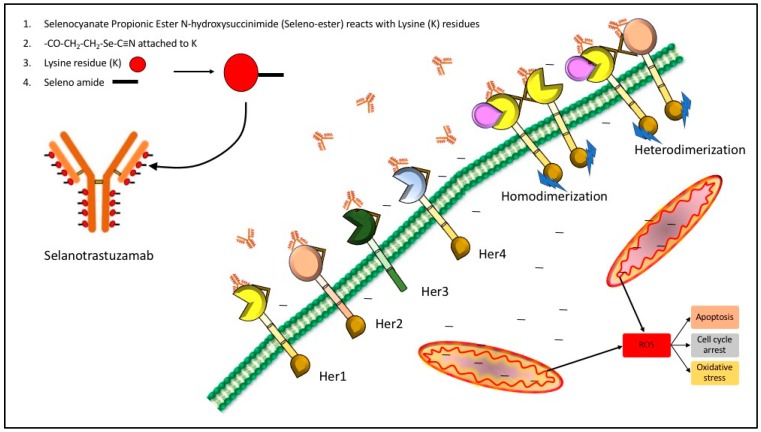
Proposed model of Selanotrastuzamab action. The promiscuity of epidermal growth factor receptor (EGFR) family binding and interaction may be one possible explanation for the observed cytotoxicity in the triple negative breast cancer cells. It is possible for Selanotrastuzamab to interact with other family members, allowing for endocytosis of a critical amount of selenium-bound trastuzumab. The selenium might then become liberated within the cytosol and enter the mitochondria where it disrupts cancer cell metabolism and produces reactive oxygen specieis (ROS).

The data from the experiments shown here and past data collections presented elsewhere, show that redox selenium may be easily attached to any “naked” monoclonal antibody or protein that has a known targeting receptor and that the selenium ADC is very likely to be more cytotoxic to the receptor containing cell than the non-receptor containing cell. This is congruent with other studies of redox active selenium compounds being more toxic to cancer cells than the corresponding normal cell. The key questions our laboratory is currently investigating are: (1) How does selenium conjugated to TZ (not observed by Western blots) kill the TNBC cells, and (2) Why are the normal cells not affected? The possibilities we are exploring are herein proposed. Here, for TZ and Se-TZ, the receptor is assumed to be an HER2 epitope but not HER2 per se (sic) as it was not expressed in any of the three cell lines. While HER2 is very low in expression, it is not entirely absent [44] and perhaps there is a minimum threshold at which selenium will affect cancer cells based on their abnormal metabolic requirements. Additionally, a review by Kennedy et al., [45] discusses the promiscuity of the EGFR family, suggesting that interactions between distantly-related receptor tyrosine kinases occur much more frequent than initially expected. This would suggest that Se-TZ binding may be occurring at other EFG receptors. Since EGFR is well-established as being up-regulated/over expressed in breast cancers with amplification and mutations especially prevalent in >60% of TNBC cases [46], it is one possibility for the increased toxicity observed in TNBC cells versus the normal cells. This proposed model is illustrated in Figure 15.

With respect to conjugating selenium to BV, we were interested in the efficacy of a selenium antibody conjugate against a ligand. VEGFs and their receptors play key roles in many cancers, where they promote the growth of tumor vasculature, promoting metastatic potential [47]. Thus, disrupting signaling by VEGF or utilizing it as a vehicle for selenium transport would demonstrate strong potential for therapeutic use if cytotoxicity to normal cells was decreased and patient survival was increased. BV is considered the number 1 selling drug globally and is FDA approved in the United States for colon and rectal cancers. Yet the lack of significant increase in life expectancy and debilitating side effects caused the FDA to revoke its use for breast cancer. Our data demonstrated that Se-BV was effective in killing TNBC cells but not the normal HME-50-5E cells when the cell lines exhibited relatively comparable levels of VEGFA. In this case, the mechanism of action is not yet known, and we are exploring possible scenarios that investigate expression levels of VEGFRs on the TNBC cells and normal breast epithelial cells as our starting point.

Regardless of the as yet undiscovered mechanism, selenium ADCs would seem to offer a better, more efficient method of targeting cancer cells, while being less toxic to normal bystander cells, as it is in fact an essential cell nutrient. Thus, exploiting the metabolic differences between cancer and normal cells may be a more effective mechanism of killing the cancer while reducing the toxic side effects of systemic DNA attacks. No release of the redox selenium from the ADC is needed to be toxic as indicated by the in vitro CL assays generating CL. Dose and time are the only keys to a potentially better in vivo cancer treatment as low dose selenium exposure to bystander cells will use the selenium from the ADC as a nutrient for the synthesis of selenocysteine for those 25 selenocysteine human proteins. Future experiments will likely reveal the clinical value of redox selenium ADCs towards TNBC and other cancer treatments.

## 4. Materials and Methods

### 4.1. Materials

DMEM high glucose (Catalog# 11965-092), Fetal Bovine Serum (Catalog# 10082-147), 1% Penicillin-Streptomycin (Catalog# 15-140-122) were purchased from Life Technologies, Gibco (Carlsbad, CA, USA) pH test strips (Catalog# 8882-1) were purchased from Ricca Chemical Company (Arlington, TX, USA). PVDF membranes (Catalog# 1620177), Non-Fat Milk Protein (Catalog# 1706404), 3–20% Tris-Glycine Polyacrylamide Gel (Catalog# 456-1096) and 2× native PAGE sample buffer (Catalog# 161-0738) were purchased from BIO-RAD (Hercules, CA, USA). Mammary Epithelial Cell Media (Catalog# 50-306-176) was purchased from PromoCell (Heidelberg Germany). Trypsin (Catalog# 30-2101) was purchased from ATCC (Manassas, VA, USA). Rabbit Anti-Mouse IgG H&L HRP (Catalog# ab6728) was purchased from Abcam (Cambridge, UK). 0.22 µM filter (Catalog# SLGV033RS), Anti-β-Actin Antibody (Catalog# MAB1501) and Accutase (Catalog# SCR005) from EMD Millipore (Burlington, MA, USA). Corning^®^ cell culture 75 cm^2^ (Catalog# CLS430641) and 25 cm^2^ (Catalog# CLS430639) vented cap flasks, Corning^®^ Costar^®^ TC-Treated Multiple Well Plates (Catalog# CLS3527), Corning^®^ Costar^®^ TC-Treated Multiple Well Plates (Catalog# CLS3548), Sodium selenite (Catalog# 214485), MTT (Catalog# M2128), Superoxide Dismutase (Catalog# S7571), Catalase (Catalog# C1345-1G), Dihydroethidium (Catalog# D7008), Tetrahydrofuran (Catalog# 34865) were purchased from Sigma-Aldrich (St. Louis, MO, USA). RIPA lysis buffer (Catalog# 89900), VEGF (JH121) monoclonal antibody (Catalog# MA5-13182), SuperSignal™ West Femto Maximum Sensitivity Substrate (Catalog# 34095), Gamma Globulin (Catalog# 23212), BCA assay kit (Catalog# 23225), MitoTracker™ Red CMXRos (Catalog# M7512), Annexin V-FITC Apoptosis Detection Kit (Catalog# BMS500FI-300) were purchased from ThermoFisher Scientific (Waltham, MA, USA). Dialysis tube (Catalog# 132542) was purchased from Spectrum Labs (Waltham, MA, USA). Bovine Serum Albumin (Catalog# BP1600-100) was purchased from Fisher Scientific. HER2/ErbB2 (44E7) mouse mAb (Catalog# 2248S) was purchased from Cell Signaling Technology (Danvers, MA, USA). Protein ladders of size of 10–245 kDa was purchased from (GeneTex, Irvine, CA, USA, Catalog# GTX50875).

### 4.2. Methods

**Figure A5:**
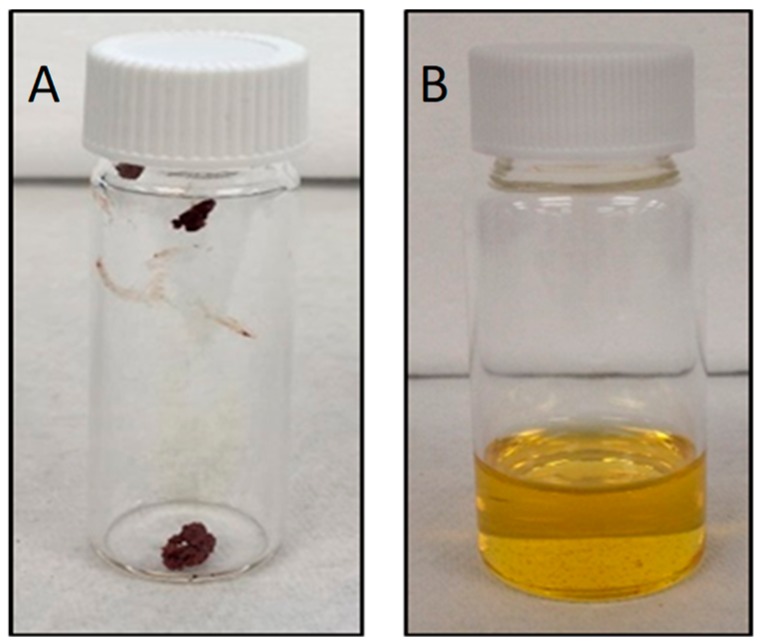
(**A**) Selenocyanate Propionic Ester of *N*-hydroxysuccinimide (Bolton-Hunter Reagent). (**B**) Unsaturated Solution of Se-Ester dissolved in Tetrahydrofuran at a concentration of 20 mg/mL.

A modified selenium Bolton-Hunter Reagent was synthesized by Eburon Organics N.V., Belgium from *N*-Hydroxysuccinamide and 3-Selenocyanoproprionic acid and was used as received. The selenium complex color is dark red (Figure A5). TZ and BV were obtained through collaborative efforts with Everado Cobos, Director, the TTUHSC Southwest Cancer Center Clinic, Lubbock, TX, USA.

**Figure 16:**
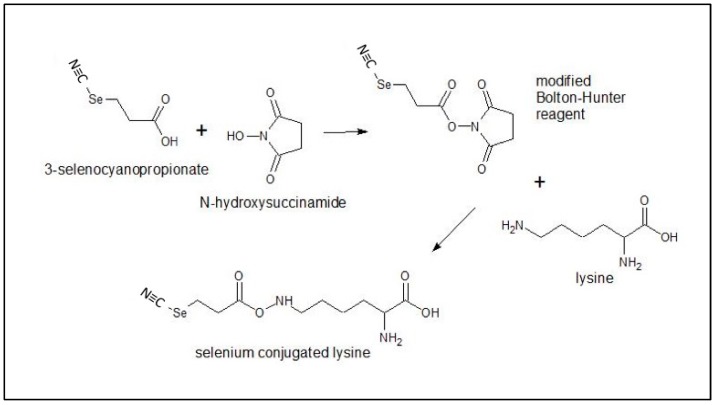
Generation of the modified Bolton-Hunter reagent for conjugation of selenium to lysine.

Selenotrastuzumab (Se-TZ) and Selenobevacizumab (Se-BV) were synthesized in-house using the selenium-modified Bolton-Hunter reagent. The covalent attachment of a redox active selenide to targeting proteins and monoclonal antibodies with the Se-Bolton-Hunter reagent is only a recent development [48]. The Se-modified Bolton-Hunter reagent was subsequently used to attach catalytic redox Se to lysine residues of TZ and BV. Figure 16 schematically shows the reactions.

#### 4.2.1. Conjugation of Redox Se to Monoclonal Antibodies

TZ and BV were diluted to a concentration of 3 mg protein/mL from stock solutions of 25 mg/mL and 21 mg/mL, respectively, with 0.05 M sodium borate buffer (pH 8.5) at 4 °C. The Se-ester was dissolved in tetrahydrofuran at a concentration of 20 mg/mL (and 2.5 mL (20 mg/mL) of the seleno-ester in tetrahydrofuran (THF) was added slowly into 10 mL of the diluted TZ and BV with the glass vials kept on ice. The reaction forming seleno-containing amides with BV and TZ lysine residues was carried out for 72 h at 4 °C. Dialysis tubing in cold 0.05 M phosphate buffer (pH 7.4) was soaked for 24h at 4 °C prior to use. After reaction incubation for 72 h, TZ and BV from their glass vial were transferred to dialysis tubing (MWCO 50,000) and were placed in the refrigerator for another 72 h in dialysis buffer. The dialysis buffer (0.05 M phosphate buffer, pH 7.4) was changed 3–4 times the first day and then twice daily thereafter. For dialysis, 10 mL of the antibody in 2 L of dialysis buffer was used. After 72h, TZ, BV, Se-TZ and Se-BV contained within the dialysis tubing was taken out and rinsed with sterile water, and the antibodies were then drawn from the dialysis tubing with a 16-gauge needle and syringe. The antibodies were filtered into sterile scintillation vials using a 0.22 µM filter. BV, TZ, Se-BV and Se-TZ were stored prior to use at 4 °C. The scintillation vials containing antibodies were covered with aluminum foil to avoid light. The mAbs were kept on ice during handling. BV, TZ, Se-BV and Se-TZ were run through the Attune™ NxT Flow Cytometer (ThermoFisher Scientific) to detect any background fluorescence for subsequent fluorescent assays.

#### 4.2.2. Analysis of Se-TZ and Se-BV

All antibodies were sent to TraceAnalysis, Inc. (Lubbock, TX, USA) for elemental selenium analysis performed by ICP-MS.

#### 4.2.3. Detection of Superoxide in Vitro by BV, Se-BV, TZ and Se-TZ

Lucigenin (Bis-*N*-Methylacridinium Nitrate) was used to detect superoxide in a chemiluminescent (CL) assay consisting of PBS pH 7.4 and reduced GSH. The amount of superoxide generated is proportional to the chemiluminescent light detected using a Turner TD-20e Luminometer (Turner Designs Inc., Mountain View, CA, USA), connected to a circulating water bath. The source of electrons for superoxide is from oxidized GSH catalyzed by selenite and selenides. Chemiluminescence (CL) measurements of BV, TZ, Se-BV and Se-TZ were performed in triplicates by adding 100 µL of BV, TZ, Se-BV or Se-TZ to 500 µL of the chemiluminescent cocktail at 37 °C with integrations of 30 s over 12.5 min, N = 25.

#### 4.2.4. Cell Culture

MDA-MB-468 cells were obtained from ATCC. HME50-5E cells were derived from tissue obtained by surgical resection that spontaneously immortalized as previously described (Shay et al., 1995). MDA-MB-468 cells were cultured in 75 cm^2^ tissue culture flasks and maintained in high glucose Dulbecco’s Modified Eagle Medium (DMEM) media supplemented with 10% fetal bovine serum and 1% Penicillin-Streptomycin. The cells were incubated for 2 to 3 days at 37 °C under humid conditions in a 5% CO_2_ incubator (ThermoFisher Scientific). The growth media was changed twice weekly. Cells were grown to 75–85% confluence then washed with 1× phosphate buffer saline (PBS), trypsinized with 5 mL of 0.25% (v) trypsin with 0.03% EDTA, diluted with fresh media, and counted using a Beckman Coulter ViCell (Beckman Coulter Inc., Brea, CA, USA, Model VI-CELL SGL).

HME50-5E cells were also cultured in 25 cm^2^ tissue culture flask and maintained in Mammary Epithelial Cell Media and were incubated for 7 to 8 days at 37 °C under humid conditions in a 5% CO^2^ incubator. The media was changed every other day. Cells were grown to 60–70% confluence, then washed with ice cold 1× phosphate buffer saline (PBS), trypsinized with 5 mL of Accutase, diluted with fresh media, and counted using a Beckman Coulter ViCell (Beckman Coulter, Inc., Model VI-CELL SGL).

Optimization of the initial cell density for treatment experiments was determined from log growth cell curves in 48-well plates performed by seeding 5000; 10,000; 20,000; 40,000 and 100,000 cells/well and cell growth was plotted against time for 7 days. This was done to determine and assure a logarithmic growth of cells throughout the latter experimental time period.

#### 4.2.5. Cell Treatments

All experiments were performed under aseptic conditions. Exponentially growing cells were harvested from flasks, counted by Trypan Blue exclusion, and plated into 48-well plates at the determined optimal seeding density of 40,000 cells/well (MDA-MB-468, HME50-5E) per well. Cells were allowed to grow for 5 days post-seeding prior to day 0 of treatment. Media was changed on day 3 post-seeding and treatments were begun with the addition of fresh culture media.

TZ and BV were the experimental controls for Se-TZ and Se-BV cell treatments, respectively. Sodium selenite was used as a positive control for cell-death. Control cells were seeded and treated as indicated with 1X PBS. TZ, Se-TZ, BV, Se-BV or selenite added to the MDA-MB-468 and HME50-5E cells.

#### 4.2.6. Photographic Assessment of Cellular Morphology

A phase-contrast microscope, EVOS XL Core (Life Technologies) was used for photographing cell morphological changes due to selenite, TZ, Se-TZ, BV or Se-BV treatments.

#### 4.2.7. Cell Viability Measured by Trypan Blue Exclusion

Cell viability from the control and experimental treated cells was determined using a Beckman Coulter Vi-Cell Viability Analyzer (Beckman Coulter, Inc., Model VI-CELL SGL) with cell viability based on Trypan Blue cell exclusion.

Forty-thousand cells/well were seeded in 48-well flat-bottom plates, cell volumes were adjusted with media and cells were allowed to grow for 5 days prior to treatments. Media was changed on day 3 post seeding and just before treatments. Cells were treated with Se-BV (2 µg Se from 30.7 µg protein) and Se-TZ (2 µg Se from 26.2 µg protein) in triplicate for 7 days. Cells were also treated with selenite (2 µg Se). Experimental cells were concentration- and time-dependent treated in comparison to native antibody BV (30.7 µg protein) and TZ (26.2 µg protein).

On days 0 through 7 of treatments; 500 µL of media from each well was collected. The cells were rinsed with 500 µL of 1× PBS and 200 µL of 0.025% trypsin-EDTA was added to harvest adherent cells. After 5 min of incubation, the remaining trypsin solution was added to the cell collections. Cells were analyzed with the Beckman Coulter Vi-Cell Viability Analyzer with the following settings:

Cell type: BrCa cells
Minimum diameter (microns): 12
Maximum diameter (microns): 50
Cell brightness (%): 85
Cell sharpness: 100
Viable cell spot brightness (%): 65
Viable cell spot area (%): 5

#### 4.2.8. Measuring Cell Proliferation Using MTT Assay

Cells were treated on day 5 post-seeding with Se-BV (2 µg Se from30.7 µg protein) or Se-TZ (2 µg Se from 26.2 µg protein) in triplicates for 6 days. Cells were also treated with equivalent BV or TZ and selenite (2 µg as Se). Blank wells without cells contained complete growth media (Phenol red free DMEM high glucose + 10% FBS). MTT was dissolved in phenol red-free media, 5 mg/mL was passed and sterilized through a 0.22 µM filter inside the cell culture hood. The tube containing the MTT solution was wrapped with aluminum foil and used immediately. To each well, 10% (*v*/*v*), 50 µL MTT (5 mg/mL) was added and incubated at 37 °C for 3 h. Following incubation with MTT, the Formazan solubilization solution [acidified isopropanol (0.1 N HCl) with 10% Triton X-100] equal to the original volume of media (500 µL) was added in each well. Dissolved Formazan in each well was determined by a Cytation 3 plate reader (BioTek, Winooski, VT, USA) at 570 nm with baseline subtraction of 690 nm absorption to assess cell viability. The control cells were considered to be 100% viable. The % cell viability was calculated using the following Equation (1).

(1) $$\mathrm{Cell}\;\mathrm{Viability}=\left[\frac{\left(\mathrm{Experimental}\;\mathrm{Absorbance}\right)}{\left(\mathrm{Control}\;\mathrm{cells}\;\mathrm{Absorbance}\right)}\right]\times 100$$

#### 4.2.9. MitoTracker^®^ Red and Annexin V Staining

Into 48-well flat-bottom plates 40,000 cells/well were seeded and were allowed to grow for 5 days prior to treatment. Media was changed on day 3 post-seeding and volume was adjusted before treatment. Cells were treated (on day 5 post seeding) with Se-BV (2 µg Se and 30.7 µg protein) and Se-TZ (2 µg Se and 26.2 µg protein). Equivalent protein concentrations of native BV or TZ, or selenite (2 µg Se/well) were added. Sets of unstained control cells and cells for staining were prepared for flow cytometry. For cell necrosis control (double-negative), cells were treated with 50 µL of 0.01% Triton X-100 for 30 min prior to staining or 200 µL of H_2_O_2_ for 24 h prior to staining. For single staining controls, appropriate wells were treated separately with the dyes (Annexin V− 488 cells were treated with apoptosis inducer 5 µL of 1 mM Sutent; untreated with MitoTracker^®^ Red). For double staining controls (double-positive), appropriate wells were treated with both dyes of the same volume and were set aside in 2 mL Eppendorf tubes. Each treatment was repeated in triplicate.

The 10 mM stock solution of MitoTracker^®^ Red dye was prepared by adding 9.4 µL DMSO to a vial of MitoTracker^®^ Red dye. A 10 µM working solution of the MitoTracker^®^ Red dye was prepared by pipetting 1 µL of 10 mM MitoTracker^®^ Red stock solution into 1000 µL of DMEM high glucose phenol red free cell media. 2 µL of 10 µM MitoTracker^®^ Red working solution was added to each well (500 µL) and stained for 30 min at 37 °C in an incubator with a humid atmosphere of 5% CO_2_. After 30 min of incubation, the media was collected in a sterile 2 mL Eppendorf tube. The cells were washed with 500 µL of 1× PBS and collected in Eppendorf tubes. Cells were trypsinized using 200 µL of 0.025% trypsin-EDTA and centrifuged. Cells were resuspended in 100 µL of 1× Annexin-binding buffer and this suspension was transferred into 96-well flat-bottom plates. Four µL of Annexin V dye was added to each well and the 96-well plate was wrapped in aluminum foil. The cells were incubated for 15 min at 37 °C in an incubator with a humid atmosphere of 5% CO_2_. After the incubation, 100 µL of 1× Annexin-binding buffer was added. The plate was placed immediately on ice. The stained cells were analyzed by flow cytometry using the Attune™ NxT Flow Cytometer (ThermoFisher Scientific). The laser lines were set to BL1 (200 nm) and YL1 (336 nm). Voltage for Forward Scatter was set to 120 V and Side Scatter to 290 V.

#### 4.2.10. Detection of Intracellular Superoxide Generation

MDA-MB-468 and HME50-5E cells were seeded at a density of 2 × 10^5^ cells/well in 24-well flat bottom plates. After 48 h, DMEM high glucose media without phenol red supplemented with FBS was added. Fifty units/well of Superoxide dismutase (SOD) from bovine erythrocytes and 100 units/well of Catalase from bovine liver in media were added to all wells. All cells-control, selenite (10 µg Se), BV (153.7 µg protein), Se-BV (10 µg Se in 153.7 µg protein), TZ (131.1 µg protein), Se-TZ (10 µg Se in 131.1 µg protein) were treated for 30 min. DHE was added at a final concentration of 10 µM/well. Cells were visually assessed under RFP using the EVOS FL Auto Cell Imaging System (ThermoFisher Scientific) or Cytation 3 plate reader (BioTek).

#### 4.2.11. BCA Assay for Protein Determination of mAbs

Total protein content of BV, TZ, Se-BV or Se-TZ after dialysis was determined using the Bicinchoninic acid (BCA) assay kit. Ten µL of BV, TZ, Se-BV or Se-TZ were diluted in 90 µL of phosphate-buffered saline followed by 2 mL of working reagent. After the addition of working reagent, the samples were incubated in cuvettes at 37 °C in a water bath for 30 min. One empty 96-well plate was kept in the incubator. After 30 min of incubationm both the cuvettes and 96-well plate were cooled down to room temperature. Two-hundred-and-twenty-five µL of the colored solution from cuvette was added to each of the three wells in the 96-well plate. Absorbance was read using a Cytation 3 plate reader (BioTek) at 562 nm absorption. Protein concentrations were determined from a standard curve prepared from a serial dilution of bovine gamma globulin. Protein concentrations were also determined using a Cytation 3 plate reader (BioTek).

#### 4.2.12. PAGE Under Denaturing Conditions

Concentrations of 5, 10 and 20 µg of each antibody-BV, TZ, Se-BV and Se-TZ in 6× Laemmli buffer were mixed and boiled for 5 min at 95 °C. Protein ladders of 10–245 kDa were used to determine the denatured antibody molecular weights. Twenty µg of bovine gamma globulin and purified human IgG were loaded as controls. The treated mAbs were centrifuged for 1 min at 11,000× *g* and the total volume of the samples was loaded into a BIO-RAD miniprotean 14 well comb (1.5 mm) 8% polyacrylamide gel for electrophoresis. Initially, the samples were run at 50 V for 5 min and then the voltage was increased to 90 V for 90 min. The SDS-PAGE gel was stained with Coomassie Blue R-250 for 15 min and then destained with Coomassie blue destaining solution. The gels were then transferred to water and photographs were taken under a Coomassie Blue filter using a LI-COR (Model: 2800, S/N OFC-0786, LI-COR, Inc., Lincoln, NE, USA).

#### 4.2.13. PAGE Under Non-Denaturing Conditions

Five and 10 micrograms of protein from BV, TZ, Se-BV and Se-TZ in 12 µL, were mixed with 6 µL of 2× native PAGE sample buffer. Protein ladders 10–245 kDa were used to verify the molecular weights of the antibodies. Twenty µg of bovine gamma globulin and a purified human IgG were also used as loading controls. Samples were run on a gradient of 3–20% Tris-Glycine Polyacrylamide Gel. Initially, the samples were run at 50 V for 5 min and then the voltage was increased to 160 V for 1 h. The gel was stained with Coomassie Blue R-250 for 15 min and destained with Coomassie Blue destaining solution for 20 min, then transferred to water. The gels were then transferred to water and photographs were taken under a Coomassie Blue filter using a LI-COR (Model: 2800, S/N OFC-0786, LI-COR, Inc.).

#### 4.2.14. Western Blotting

One million MDA-MB-468 and HME50-5E cells were allowed to attach to T-25 flasks overnight. The following day cells were treated with 2 µg as Se, 26.22 µg as protein of Se-TZ or 2 µg of Se and 30.75 µg protein of Se-BV and incubated for 3 days. Control cells were treated with 1× PBS using the same experimental time.

On day 3 of treatment, cultured cell fluid was collected in a 15 mL centrifuge tube and cells were washed with ice cold 1× PBS and collected in the same tube. The tube was centrifuged at 4000× *g* for 4–5 min and the media was aspirated using a Pasteur pipette. Two-hundred µL of RIPA lysis buffer was added to the tube and everything was transferred to the T25 flasks. The T25 flasks were kept at −80 °C for 1 day. Cancer cell protein lysates’ yield was better when the flask was thawed. To collect the HME50-5E cell lysates, the flasks were broken with a hammer and cells were scrapped and kept on ice for 5 min. The HME50-5E lysates were passed through a 20 G needle and kept on ice for 5 min. All cell lysates were kept on an inverter for 15 min at 4 °C and centrifuged at 12,000× *g* for 15 min at 4 °C. Total protein concentration in the cleared lysate was determined by the bicinchoninic acid (BCA) assay according to manufacturer’s instructions. BT-474 cell lysate was used as a positive loading control for HER2. Forty µg of total protein was separated on 8% denaturing polyacrylamide gels and transferred to PVDF membranes by electroblotting. Membranes were blocked for 1 hour with Tris-buffered saline containing 0.05% Tween-20 (TBST) and 5% non-fat milk protein for HER2 or 5% bovine serum albumin fatty acid free for VEGF. They were incubated overnight with HER2/ErbB2 (44E7) mouse mAb diluted 1:1000 in TBST or with VEGF (JH121) monoclonal antibody diluted 1:100 in TBST and anti-β-actin antibody diluted 1:1000 in TBST containing 1% non-fat milk protein or 1% bovine serum albumin. The next day, the membrane was washed three times for 45 min in TBST, incubated for 1 h with horse-radish peroxidase conjugated with rabbit anti-mouse IgG diluted 1:10,000 in TBST, and washed once in TBST for 15 min. Bound antibody complexes were visualized using SuperSignal™ West Femto Maximum Sensitivity Substrate.

## 5. Conclusions

Triple negative breast cancer cells (TNBC) presently have no targeting therapies as TNBC cells lack the over expression of HER2, PR and ER, so therapy is limited to chemotherapy and the prognosis for TNBC patient treatments is often poor. Having discovered that covalent redox selenium generating superoxide attached to TZ, Se-TZ, is much more effective against in vitro cell growth of a Herceptin^®^ resistant cell line, JIMT-1 [32], redox selenium was similarly attached to TZ, BV and was used in vitro against the TNBC cell lines MDA-MB-468 and MDA-MB-231. An immortalized more normal breast epithelial cell line, HME50-5E was likewise treated.

TZ and BV were successfully labeled with redox selenium following a modified Bolton-Hunter chemistry and were shown to generate superoxide both in vitro in a CL assay and in an intracellular assay using dihydroethidium. Se-TZ, Se-BV and selenite were both shown to disrupt the cellular membrane photographically and by Trypan Blue absorption. Apoptosis was shown to occur in both TNBC cell lines treated by Se-TZ, Se-BV and selenite in an MTT assay and by flow cytometry using MitoTracker^®^ Red and Annexin V staining. Concurrent and parallel experiments with the normal breast epithelial HME50-5E cells reveal much less to almost no cell damage or toxicity from Se-TZ or Se-BV. Selenite was to different degrees, toxic to all cells. TZ and BV were not comparatively effective in altering any cell morphology, nor revealing superoxide generation in vitro or intracellularly. TZ and BV did not significantly induce apoptosis in any cell line, demonstrating why TZ is not used against TNBC cells and why BV, which was initially approved for breast cancer therapy by the FDA in 2008, was disallowed for BC treatment in 2011 as it was deemed by the FDA to be ineffective and posed serious side effects [49].

The overriding consideration for this study and other in vitro therapeutic applications of redox selenium is the simplicity with which almost any selenium targeting conjugates can be formulated and how effectively they inhibit cell proliferation by internally generating superoxide and other ROS while at the same time demonstrating significantly less cytotoxicity to normal cells.

In summary, the goal of the study was to investigate efficacy of selenium to kill TNBC cells by delivery of Se via different targets; a ligand (VEGF) and a transmembrane protein receptor (HER2). Furthermore, we were interested in determining whether the metabolic differences inherent between breast cancer cells and normal mammary epithelial cells would attenuate side effects of the Se-immunoconjugates. These in vitro studies demonstrated that the selenium immunoconjugates, Se-BV and Se-TZ decrease cell viability and proliferation of Triple Negative Breast Cancer (TNBC) cells, MDA-MB-468 and MDA-MB-231. 

In our current study, selenium acted as the cytotoxic agent with two monoclonal antibodies serving the dual role of acting as a carrier for selenium and as targeting agents to deliver selenium to the TNBC cell lines. Organic diselenides, as shown by Chaudière et al. [16], and selenocyanates and isoselenocyanates as shown by Crampsie et al. [50], may continuously undergo the redox cycling and generate superoxides from reduced thiols. Here, the organic selenocyanate (RSeCN) was attached to Bevacizumab and Trastuzumab to investigate whether these current anticancer mAbs can be re-purposed and utilized to target TNBC cell lines. Selenocyanate reacts with thiols and generates selenoate anions (RSe^−^) via a selenylsulfide intermediate (RSe-SG) as illustrated in the reactions below:

RSeCN + GSH → RSeSG + HCN(2)

RSeSG + GSH → RSe- + GSSG(3)

RSe^−^ + O_2_ →O_2•_− + RSe (This is a RSe radical) (4)

The selenoate anions (RSe^−^) subsequently undergo redox cycling and produce superoxide. The presence of superoxide is the most likely reason for the catastrophic effects of Se observed in the cells regardless of introduction.

The last important observation from this work is that in each approach to selenium conjugation and delivery to the cells, the selenium was being internalized (again, evidenced by DHE ROS formation). How the selenium is being internalized is the focus of ongoing research projects. Development of a drug-carrier targeting approach, by using a dietary nutrient, likely results in reduced systemic drug toxicity towards normal cells making this study different. Furthermore, the study demonstrates that the method of delivery of redox selenium into the TNBC cells (thought to lack specific markers for targeting strategies) can be effectively piggy-backed onto current clinical therapies while demonstrating less cytotoxicity in the normal cells. These studies have profound implications for future drug design and efficacy with less side effects, impacting both treatment outcomes and quality of life in patients with TNBC and other cancers.

Our results yield proof-of-concept for consideration of potential therapeutic possibilities underlying the design of targeted cancer therapy with seleno-immunoconjugates. Small redox active selenium compounds and the more than 70 ADCs in various phases of clinical trials may be clinically effective in cancer treatment. The overriding consideration following the experimental results for this therapeutic technology is the simplicity with which redox selenium targeting conjugates can be formulated and how effectively they inhibit cell proliferation by generating superoxide and other ROS while demonstrating significantly less cytotoxicity to normal cells.

## Figures and Tables

**Table 1:** Selenium and protein concentration of native and selenium immunoconjugates following dialysis.

	TZ	Se-TZ	BV	Se-BV
Selenium Concentration (mg/L)	<0.0200	88.00	<0.0200	81.3
Protein Concentration (mg/mL)	2.82	2.32	2.73	2.53
Selenium Concentration/ mg of protein (µg/mg)	<0.007	38.00	<0.007	32.12

**Table 2:** ANOVA Results for Cell Viability with Se-TZ Treatment for MDA-MB-468 Cells.

Experiment Name	Treatments	Treatment Day	F Value	*p* Value
Trypan Blue	Control, Selenite, TZ and Se-TZ	Day 1	F (3,8) = 4	0.052
Trypan Blue	Control, Selenite, TZ and Se-TZ	Day 1	F (3,8) = 12.52	0.0022
Trypan Blue	Control, Selenite, TZ and Se-TZ	Day 2	F (3,8) = 105.45	0.0000009
Trypan Blue	Control, Selenite, TZ and Se-TZ	Day 3	F (3,8) = 164.18	0.00000015
Trypan Blue	Control, Selenite, TZ and Se-TZ	Day 4	F (3,8) = 58.78	0.0000085
Trypan Blue	Control, Selenite, TZ and Se-TZ	Day 5	F (3,8) = 427.72	0.00000000361
Trypan Blue	Control, Selenite, TZ and Se-TZ	Day 6	F (3,8) = 242.71	0.0000000341
Trypan Blue	Control, Selenite, TZ and Se-TZ	Day 7	F (3,8) = 5.5	0.0241

**Table 3:** ANOVA Results for Cell Metabolism with Se-TZ Treatment for MDA-MB-468 Cells.

Experiment Name	Treatments	Treatment Day	F Value	*p* Value
MTT	Control, Selenite, TZ and Se-TZ	Day 1	F (3,8) = 2.41	0.142
MTT	Control, Selenite, TZ and Se-TZ	Day 2	F (3,8) = 0.19	0.9018
MTT	Control, Selenite, TZ and Se-TZ	Day 3	F (3,8) = 2.13	0.175
MTT	Control, Selenite, TZ and Se-TZ	Day 4	F (3,8) = 6.19	0.013
MTT	Control, Selenite, TZ and Se-TZ	Day 5	F (3,8) = 45.69	0.000022
MTT	Control, Selenite, TZ and Se-TZ	Day 6	F (3,8) = 58.78	0.00000857

**Table 4:** ANOVA Results for Cell Viability with Se-BV Treatment for MDA-MB-468 Cells.

Experiment Name	Treatments	Treatment Day	F Value	*p* Value
Trypan Blue	Control, Selenite, BV and Se-BV	Day 0	F (3,8) = 6.24	0.0173
Trypan Blue	Control, Selenite, BV and Se-BV	Day 1	F (3,8) = 15.43	0.0010909
Trypan Blue	Control, Selenite, BV and Se-BV	Day 2	F (3,8) = 71.05	0.00000415
Trypan Blue	Control, Selenite, BV and Se-BV	Day 3	F (3,8) = 33.96	0.000067
Trypan Blue	Control, Selenite, BV and Se-BV	Day 4	F (3,8) = 69.1	0.000004
Trypan Blue	Control, Selenite, BV and Se-BV	Day 5	F (3,8) = 50.47	0.000015
Trypan Blue	Control, Selenite, BV and Se-BV	Day 6	F (3,8) = 40.38	0.000035
Trypan Blue	Control, Selenite, BV and Se-BV	Day 7	F (3,8) = 2.79	0.1096

**Table 5:** ANOVA Results for Cell Metabolism with Se-BV Treatment for MDA-MB-468 Cells.

Experiment Name	Treatments	Treatment Day	F Value	*p* Value
MTT	Control, Selenite, BV and Se-BV	Day 1	F (3,8) = 0.41	0.7479
MTT	Control, Selenite, BV and Se-BV	Day 2	F (3,8) = 0.15	0.9278
MTT	Control, Selenite, BV and Se-BV	Day 3	F (3,8) = 0.1	0.9594
MTT	Control, Selenite, BV and Se-BV	Day 4	F (3,8) = 2.02	0.1894
MTT	Control, Selenite, BV and Se-BV	Day 5	F (3,8) = 29.58	0.000111
MTT	Control, Selenite, BV and Se-BV	Day 6	F (3,8) = 69.38	0.0000045

## Abbreviations

BV	Bevacizumab
BCA	Bicinchoninic Acid
CL	Chemiluminescence
DCC	*N*,*N*′-dicyclohexylcarbodimide
DHE	Dihydroethidium
ECL	Enhanced chemiluminescence
EGFR	Epidermal growth factor receptor
ESR1	Estrogen receptor α
FDA	Food and drug administration
HER2	Human epidermal receptor 2
mAb(s)	Monoclonal antibody(ies)
MTT	Tetrazolium dye 3-(4,5-dimethylthiazol-2-yl)-2,5-diphenyltetrazolium bromide
PAGE	Polyacrylamide gel electrophoresis
PR	Progesterone receptor
ROS	Reactive oxygen specieis
Se	Selenium
Se-BV	Selenobevacizumab
Se-TZ	Selenotrastuzumab
THF	Tetrahydrofuran
TNBC	Triple negative breast cancer
TZ	Trastuzumab
VEGF	Vascular endothelial growth factor
VEGFR	Vascular endothelial growth factor receptor
